# Influence of Temperament on the Acceptance of Two Conscious Sedation Techniques in Toddlers Undergoing Dental Treatment: A Randomised Cross Over Trial

**DOI:** 10.1155/2023/6655628

**Published:** 2023-08-22

**Authors:** Palak Janiani, Deepa Gurunathan, Sivakumar Nuvvula

**Affiliations:** ^1^Department of Pediatric and Preventive Dentistry, Saveetha Dental College and Hospitals, Saveetha Institute of Medical and Technical Sciences, Saveetha University, Chennai 600077, Tamil Nadu, India; ^2^Department of Pediatric and Preventive Dentistry, Narayana Dental College and Hospital, Nellore 524003, Andhra Pradesh, India

## Abstract

**Background:**

Pediatric dentists often find it challenging to handle pediatric patients due to their fear, unease, and anxiety toward dental procedures. To address this, sedation agents such as intranasal midazolam and nitrous oxide are commonly used as pharmacological behavior management methods. A child's temperament affects their behavior in unfamiliar settings.

**Aim:**

To study the effect of child temperament on the acceptance of the nasal mask and intranasal drug administration in children undergoing dental treatment.

**Methods:**

Thirty-two anxious children aged three to five were randomly assigned to two groups. During the first visit, one group received intranasal midazolam sedation, while the other group received nitrous oxide administered through a mask. On the subsequent visit, the groups crossed over. The parent assessed the child's temperament, and the acceptance of the sedation methods was recorded. The Ohio State University Behavioral Rating Scale (OSUBRS) was employed to assess behavior during the administration of local anesthesia. Statistical analysis was carried out using the chi-square test and Mann–Whitney *U* test (*p* value <0.05).

**Results:**

Children exhibited greater acceptance of the nasal mask compared to using the intranasal route for delivering midazolam during the induction process. A significant statistical influence of temperament was observed on the acceptance of the nasal mask and the intranasal atomisation device (*p* value <0.05). The mean OSUBRS scores did not show any statistically significant differences between the sedation groups (*p* = 0.14).

**Conclusion:**

Most children demonstrated a more favorable acceptance of the nasal mask during the induction process; however, intranasal midazolam can serve as an effective alternative for anxious patients who struggle to keep the nitrous oxide mask on during the dental procedure. The adoption of these methods is influenced directly by the child's temperament.

## 1. Introduction

In clinical practice, behavior management often involves nonpharmacological methods and local anesthesia. However, certain children with behavioral challenges, such as fear and age-related dental anxiety, may require sedation to ensure safe and effective dental treatment [1]. Moderate sedation is commonly employed to address the needs of anxious children, aiming to prevent psychological distress and promote compliance during and after treatment [2]. Various routes can be used to administer sedative drugs for dental sedation. These include oral, inhalation, intravenous (IV), intramuscular (IM), and intranasal routes (IN) [3].

The acceptance of nasal masks and intranasal atomisation devices by children undergoing dental treatment is influenced by several factors. First, the nasal hood approach involves using a mask that covers the nose, delivering a mixture of nitrous oxide and oxygen through inhalation [4]. This method is generally well-received by children due to its noninvasive nature and minimal discomfort. The nasal mask provides a familiar and less intimidating experience than injections or oral medication, making it more acceptable for pediatric patients. One common problem associated with inhalation sedation in children is fear of the nasal mask and acceptance of the hood throughout the procedure [5].

On the other hand, the intranasal mucosal atomisation device (MAD) involves spraying the medication into the nasal cavity. Midazolam, ketamine, sufentanil, and dexmedetomidine are commonly administered via this device [6]. This method has gained acceptance due to its rapid onset of action and avoidance of gastrointestinal absorption [7]. Intranasal administration also eliminates the need for injections, reducing the fear and anxiety often associated with needles. The ease of application and quick delivery make it a good alternative for the sedation of children during dental procedures [8]. Nevertheless, spray use may result in adverse effects such as a bitter taste, burning sensations, or nasal discomfort [9].

The temperament of children undergoing dental treatment plays a significant role in their overall experience and cooperation during the procedure. Each child has a unique temperament, which refers to their individual behavioral and emotional characteristics [10]. Some children may exhibit fear, anxiety, or difficulty managing their emotions, which can pose challenges during dental visits. On the other hand, children with more positive temperament traits, such as adaptability and ease of soothing, may be more cooperative during dental procedures [11]. Understanding and assessing the child's temperament can help dental professionals tailor their approach to meet each child's specific needs, provide appropriate behavior management strategies and create a more comfortable and supportive environment.

To the best of our knowledge, there have been no studies thus far that have explored the association between a child's temperament and their acceptance of the method used for administering a sedative agent. Hence, this study aimed to examine the role of child temperament and its effect on the acceptance of the nasal mask and the intranasal atomisation device. The null hypothesis states that there is no difference in the acceptability between the two routes of administration and that there is no effect of temperament on the acceptability.

## 2. Materials and Methods

### 2.1. Study Design

This study was a randomized split-mouth crossover clinical trial conducted in a private university (Saveetha Dental College and Hospital, India) that underwent review and approval by the Institutional Human Ethics Committee (IHEC) of the same institution (IHEC/SDC/PEDO-2001/21/665). All parents/guardians were provided with a detailed explanation of the study's purpose, potential risks, and benefits of the treatment, and written informed consent was obtained. As per a previous study by Wilson et al. [12], a sample size of 32 participants per group was established, considering an alpha level of 0.05 and a study power of 80%. To accommodate potential dropouts, 38 children were included in the study. Children aged three to five years, who demonstrated positive behavior based on Frankl's behavior rating scale, were included in the study. These children had an American Society of Anesthesiologists (ASA) physical status I and required bilateral pulp therapy in the lower arch. Participants with breathing difficulties, a history of systemic illness, hypersensitivity to benzodiazepines, and those currently taking depressant drugs were excluded.

**Figure 1:**
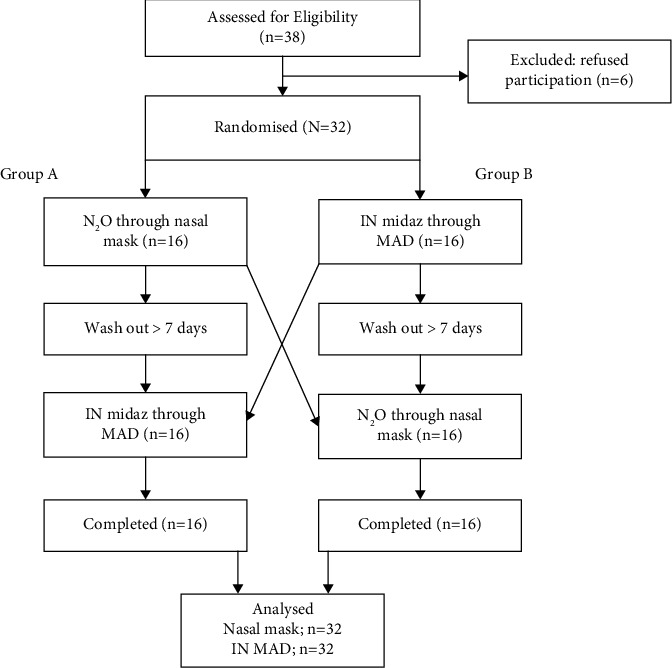
Flowchart depicting the distribution of participants (N₂O = nitrous oxide, IN = intranasal, midaz = midazolam, and MAD = mucosal atomisation device).

The child's behavior was assessed using Frankl's behavior rating scale. Only children who did not respond to conventional behavior management methods were included in the research. The preoperative instructions followed the guidelines provided by the American Academy of Pediatric Dentistry (AAPD) [13]. Participants were randomly assigned to either group A or group B on their first visit by the coin toss method. Group A participants received treatment using the nasal mask during their initial visit and subsequently received midazolam using the intranasal MAD during their second visit, while Group B experienced the reverse order of interventions (Figure 1). A washout period of seven days was considered. Since the method of administration of the two routes was easily distinguished, the operator as well as the evaluator could not be blinded.

### 2.2. Study Procedure

#### 2.2.1. Assessment of Temperament

During the initial visit, the parent was asked to classify their child according to the clinically relevant temperament types established by Thomas and Chess [14]. These temperament types consist of three categories: difficult, easy, and slow-to-warm up.  Table 1 provides a description of the distinctive characteristics associated with each temperament type.

#### 2.2.2. Administration of Midazolam via the Intranasal MAD

The participant was provided with a simplified explanation of the drug administration process. A dose of midazolam at 0.3 mg/kg (Mezolam, Neon Laboratories Ltd.) was administered using an intranasal mucosal atomizer device (MAD) (LMA MAD Nasal™, Wolfe Tory Medical Inc., USA) attached to a two mL syringe. The patient's acceptance of the drug was assessed using a scale developed by al-Rakaf et al. [8] (Table 2). After administering the sedative, local anesthesia was administered, and pulp therapy was carried out. During the administration of local anesthesia, the child's behavior was recorded using the Ohio State University Behavioral Rating Scale (OSUBRS) [15]. A one-week washout period was considered, after which the participant was recalled for the next visit.

#### 2.2.3. Administration of Nitrous Oxide via Nasal Mask

The nitrous oxide was administered by applying the nasal mask using techniques such as tell-show-do and euphemisms. Initially providing 100% oxygen for 2-3 minutes, the flow rate was determined. Subsequently, a preset combination of nitrous oxide and oxygen, ranging from 30% to 70%, was administered using a Consed (Consed International, Kerala, India) analgesia machine. The patient's acceptance of the nasal mask was assessed using a scale developed by Wood in 2010 [16] (Table 3). Local anesthesia was administered once the initial signs of sedation were observed, and behavior was recorded.

#### 2.2.4. Statistical Analysis

The analysis in this study utilized SPSS software version 23 (IBM SPSS Statistics for Windows, Version 23.0, IBM Corp). Descriptive statistics were calculated. Data analysis was performed using the chi-Square and Mann–Whitney *U* test. A significance level of *p* < 0.05 was considered statistically significant.

### 2.3. Results

A total of 32 children were included in the study, with a mean age of 5.26 ± 0.77 years. 51.4% of the children were males, and 48.6% were females.

#### 2.3.1. Acceptability of Nasal Masks and MAD

**Figure 2:**
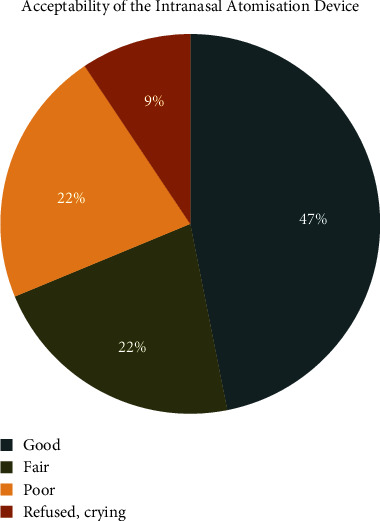
Pie chart depicting the acceptability of the intranasal atomisation device amongst the participants.

**Figure 3:**
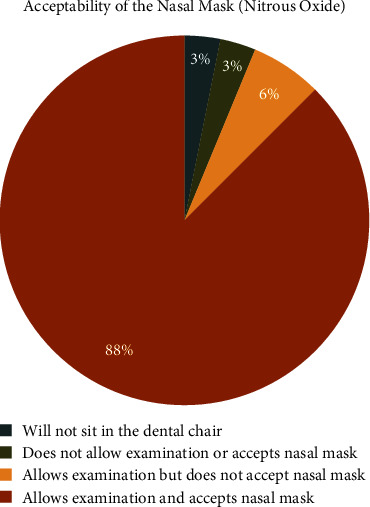
Pie chart depicting the acceptability of the nasal mask amongst the participants.

Participants' acceptability towards the intranasal atomisation device and the nasal mask is represented in Figures 2 and 3, respectively. Most children accepted the nasal mask and allowed examination, whereas only 47% showed good acceptance of the MAD.

#### 2.3.2. Association between Temperament and Acceptability during Induction

A statistically significant association was found between the children's temperament and the acceptability of the nasal mask and the intranasal mucosal atomisation device. (*p* < 0.05). Tables 4 and 5 show the association between temperament and acceptability of the MAD and nasal mask, respectively. Participants with a difficult (12.5%) and slow-to-warm up (37.5%) temperament accepted the nasal mask better than the intranasal MAD. Children with an easy temperament (50%) accepted both routes, except one participant.

#### 2.3.3. Behavior Scores

In the nitrous oxide group, 50% of the children (16) displayed a behavior score of 1, indicating quiet behavior without movement. In addition, 18.8% (6 children) scored 2, denoting crying without struggling, while another 18.8% (6 children) scored 3, representing struggling movement without crying. 12.5% (4 children) scored 4, indicating struggling movements and crying. On the other hand, in the intranasal midazolam group, 65.6% (21 children) received a score of 1, 15.6% (5 children) received a score of 2, 12.5% (4 children) received a score of 3, and 6.3% (2 children) received a score of 4. The mean scores for the two groups, as measured by the OSUBRS, are presented in Table 6. No statistically significant differences were observed between the sedation groups in terms of the mean OSUBRS scores (*p* = 0.14; Mann–Whitney *U* test).

## 3. Discussion

Effective behavior management is crucial to pediatric dentistry while providing optimal treatment for anxious children. When nonpharmacological behavior management strategies are unsuccessful in alleviating anxiety, pharmacological approaches are often considered viable alternatives [17]. The acceptance of these pharmacological approaches varies among children and is influenced by numerous factors. The present study is one of the first to explore the relationship between a child's temperament and their acceptance of the nasal mask and the intranasal mucosal atomisation device. A statistically significant correlation was established between a child's temperament and their acceptance of the nasal mask and intranasal mucosal atomisation device. Consequently, these findings led to the rejection of the null hypothesis.

Various temperament models are available, and the primary models utilized are the Behavior Style Questionnaire (BSQ) and the Emotionality-Activity-Sociability (EAS) scales [18]. The BSQ, initially introduced by Thomas and Chess, identified nine temperament dimensions. Following this, the authors categorized children into various types, including easy children, difficult children, and children with slow adaptation. The current research grouped children into various categories according to the BSQ classifications. Kaplan and Sadock discovered that 10% of children exhibit a temperament characterized as difficult, while 40% of children possess an easy temperament [19]. Our study yielded similar results, with 12.5% of children demonstrating a difficult temperament and 50% displaying an easy temperament.

The EAS tool divides temperament traits into 4 types: emotionality, activity, sociability, and shyness. Quinonez et al. conducted a study using the EAS Temperament Survey for Children to explore the potential association between temperament and trait anxiety [20]. The results suggested that children with higher emotionality and lower sociability tended to exhibit greater trait anxiety. In another study, children regarded as shy and having a negative emotionality showed an unsatisfactory acceptance to the dental treatment [21]. Due to the situational dependence of scores obtained from this tool and the lack of evidence indicating any influence of activity on children's behavior or anxiety, our study opted to utilize the BSQ scale.

Children with a difficult and slow-to-warm up temperament initially accepted the nasal mask better than the intranasal MAD. Previously, few studies have focused on evaluating the acceptability of the nasal hood and intranasal drug administration methods in children undergoing dental treatment. Srinivasan et al. observed that children displayed greater acceptance and reduced anxiety when using the nasal hood, which was attributed to its noninvasive and comfortable nature [22]. These findings align with the results of the present study. Another study by Somasundaram and Preethy examined the acceptance of intranasal midazolam spray in children receiving dental treatment [23]. The findings demonstrated high acceptance levels among children and parents, highlighting the convenience and rapid onset of action associated with the intranasal route. Intranasal administration's needle-free and noninvasive characteristics contributed to decreased anxiety levels and enhanced cooperation during dental procedures. Our study observed that 47% of the participants demonstrated acceptance of the intranasal spray. However, a larger percentage (53%) of the participants expressed some level of discomfort or resistance toward the sensation of medication being applied directly to the nose. This understanding of temperament's role can assist parents and dental professionals in selecting the most appropriate treatment approach for each child, considering their temperament characteristics.

Based on the current study's findings, children's behavior showed improvement following the administration of intranasal midazolam sedation compared to nitrous oxide during the treatment. However, this difference was not found to be statistically significant. These findings are consistent with the results reported by Panchal et al. [22]. Despite the lack of statistical significance, it was clinically observed that children who received intranasal midazolam exhibited better acceptance of the treatment. This could be attributed to the convenience of intranasal administration, which involves a single shot. In contrast, nitrous oxide necessitates a continuous flow, and it was observed that children were uncomfortable with the constant presence of the nasal mask.

A limitation of the study was the need for more behavioral variability. This may be attributed to the selection of mostly cooperative children for sedation or the automatic scheduling of children perceived as uncooperative during their initial examination for restorative treatment under general anesthesia. The parents classified the temperament of the child. However, it should be understood that parents may not recognise their child correctly or may refrain from defining him/her as difficult, potentially leading to misleading results. In addition, our study exclusively examined the administration of midazolam through the intranasal route, which is known to have a bitter taste that may have influenced the acceptance of this particular method. To address this limitation, further research is warranted to explore the use of other nonbitter sedative agents delivered via the intranasal route.

## 4. Conclusion

Children who underwent sedation using a nasal mask demonstrated higher levels of acceptance during induction when compared to the mucosal atomisation device used for intranasal administration. The temperament of the child is one of the factors that directly influences the acceptance of the sedation method; children with a difficult temperament displayed lower acceptance of the intranasal mucosal atomisation device in comparison to the nasal mask.

Clinically, dental professionals need to consider the child's temperament, comfort, and cooperation when choosing the most suitable route and method of drug administration.

## Figures and Tables

**Table 1:** Temperament types and characteristics.

Temperament trait	Characteristics
Difficult	The combination of biological irregularity, withdrawal tendencies to the new, slow adaptability, and frequent negative emotional reactions of high intensity
Easy	The combination of biological regularity, approach tendencies to the new, quick adaptability to change, and predominantly positive mood
Slow-to-warm up	Characterized by withdrawal tendencies to the new, slow adaptability, frequent negative emotional reactions of low intensity—often labeled “shy”

**Table 2:** Acceptability of intranasal administration of midazolam.

Code	Description
Good	Child accepted the drug without any refusal or resistance
Fair	Child accepted the drug administration with some verbal resistance
Poor	Child accepted the drug with some physical resistance
Refused	Child refused and drug administration was possible only after much persuasion

**Table 3:** Acceptability of nasal mask.

Code	Description
0	No cooperation at all—would not sit in the dental chair
1	Patient would sit in the chair but would not allow an intraoral examination with a mirror and would not accept the nasal mask
2	Would sit in the chair and allow an intraoral examination but would not accept the nasal mask
3	The patient sat in the chair, allowed intraoral examination, and accepted the nasal mask—very cooperative

**Table 4:** Association between temperament type and acceptance of intranasal mucosal atomisation device. (*p* < 0.05).

Temperament	Good (*n*)	Fair (*n*)	Poor (*n*)	Refused, crying (*n*)	*p* value
Difficult	0	0	2	2	<0.001^*∗*^
Easy	11	4	1	0	
Slow-to-warm up	4	3	4	1	

^*∗*^Statistically significant.

**Table 5:** Association between temperament type and acceptance of a nasal mask. (*p* < 0.05).

Temperament	Will not sit in the dental chair (*n*)	Does not allow examination or accepts nasal mask (*n*)	Allows examination but does not accept nasal mask (*n*)	Allows examination and accepts nasal mask (*n*)	*p* value
Difficult	1	0	1	2	<0.05^*∗*^
Easy	0	0	1	15	
Slow-to-warm up	0	1	0	11	

^*∗*^Statistically significant.

**Table 6:** Comparison of the mean behavior scores during the administration of local anesthesia.

Groups	OSURBS score mean ± SD
Nitrous oxide (*n* = 32)	1.94 ± 1.11
Intranasal Midazolam (*n* = 32)	1.47 ± 0.72

## Data Availability

The data used to support the findings of this study are available from the corresponding author upon reasonable request.
